# 708 Clinical Impact of a Revised Burn Resuscitation Guideline at an Adult Burn Center

**DOI:** 10.1093/jbcr/irad045.184

**Published:** 2023-05-15

**Authors:** Dominick Curry, Susan Smith, Brandon Hobbs

**Affiliations:** Orlando Regional Medical Center, Orlando, Florida; Orlando Regional Medical Center, Orlando, Florida; Orlando Regional Medical Center, Orlando, Florida; Orlando Regional Medical Center, Orlando, Florida; Orlando Regional Medical Center, Orlando, Florida; Orlando Regional Medical Center, Orlando, Florida; Orlando Regional Medical Center, Orlando, Florida; Orlando Regional Medical Center, Orlando, Florida; Orlando Regional Medical Center, Orlando, Florida

## Abstract

**Introduction:**

The American Burn Association recommends volume resuscitation at 2-4ml/kg/TBSA; however, in practice, fluid resuscitation often exceeds these goals. Additional strategies used for burn resuscitation include the use of colloid rescue and high dose ascorbic acid (HDAA). In 2018, our institution moved from a fluid resuscitation strategy which included calculating fluid requirements using 4ml/kg/TBSA and HDAA infusion for burns > 30% TBSA to a strategy that calculated fluids using a 2ml/kg/TBSA with the removal of HDAA. Additionally, the guidance for colloid rescue was updated to provide specific time points for evaluation of the need for initiation of this intervention. The primary objective of this study was to compare outcomes of the new institutional resuscitation strategy to the previous one.

**Methods:**

This was a retrospective cohort study evaluating outcomes for acute burn resuscitation between the previous institutional resuscitation guideline with the updated guideline. This study included adult patients admitted for burn injury with >20% TBSA between August 1, 2016, to September 30, 2021. The primary outcome was a composite safety endpoint which included incidence of acute kidney injury and intra-abdominal hypertension requiring intervention. Acute kidney injury was defined as a serum creatinine increase 1.5 times baseline. Statistical analyses utilized the Mann-Whitney U test for non-normally distributed continuous data and the chi-squared and Fischer’s exact test for categorical data. A p-value of < 0.05 was considered statistically significant.

**Results:**

The final analysis included 43 in the pre-group and 52 in the post-group update. Baseline characteristics between the groups were similar. The primary composite safety outcome was non-statistically significantly lower in the post-guideline update group (39.5% vs. 28.8%, p= 0.273). Total fluid volume administered (3.74 ml/kg/TBSA vs. 2.97 mL/kg/TBSA, p=0.007) and hourly urine output within the first 24 hours (1.26 mL/kg/hour vs. 0.75 mL/kg/hour, p=0.007) were significantly lower in the post-group. No differences were observed in vasopressor use (32.6% vs 36.5%, p=0.63), vasopressor duration (70.3 hours vs. 50.4 hours p=0.24), or duration of mechanical ventilation (5.1 days vs 10.4 days, p=0.113).

**Conclusions:**

The burn resuscitation guideline update resulted in significantly lower 24 hour- fluid volumes, without negative hemodynamic consequences.

**Applicability of Research to Practice:**

The comparison of two resuscitation protocols provides evidence for other institutions to help optimize their resuscitation practice

